# Successful delayed endoscopic management of dislocated hepaticogastrostomy stent with self-expanding metal stent and esophageal perforation closure

**DOI:** 10.1055/a-2570-7793

**Published:** 2025-04-09

**Authors:** André Sasse, Benjamin Christian Steuber, Volker Ellenrieder, Golo Petzold, Thomas Roland Heiduk, Ahmad Amanzada

**Affiliations:** 1 27177 Department of Gastroenterology, Gastrointestinal Oncology and Endocrinology, University Medical Center Göttingen, Göttingen, Germany

Endoscopic ultrasound-guided hepaticogastrostomy (EUS-HGS) is an emerging procedure for relieving malignant jaundice especially in palliative situations. Complications such as stent dislocation are relatively rare (3%) and are typically managed surgically 1 2 3 . There are only a few case reports of endoscopic solutions for dislocated EUS-HGS stents 4 .

We present the case of a woman who was transferred to our university medical center for surgery due to a dislocated metal stent following EUS-HGS. Initially, the patient presented with painless jaundice ( Fig. 1 ) and underwent endoscopic retrograde cholangiopancreatography (ERCP), which revealed a possibly malignant subhilar stenosis of the biliary tract. A plastic stent was placed, but the serum bilirubin level did not decrease and cholestasis persisted. The patient refused surgery and further diagnostics but agreed to undergo EUS-HGS to relieve her obstructive jaundice. After placement of the metal stent, it dislocated into the peritoneum, resulting in peritoneal bile leakage, perforation of the distal esophagus due to misplacement of the EUS-HGS stent, and pneumoperitoneum ( Fig. 2 ).

**Fig. 1:**
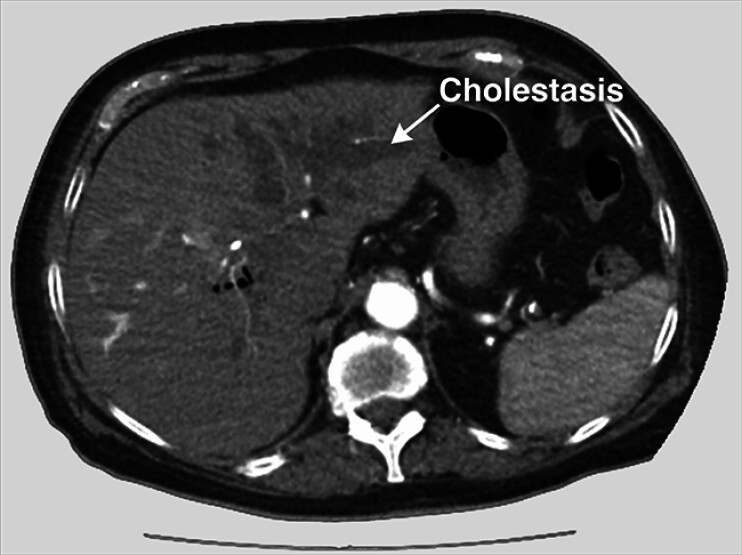
Persistent cholestasis of the left biliary system after the placement of plastic stents via endoscopic retrograde cholangiopancreatography and suspected malignant subhilar bile duct stenosis.

**Fig. 2:**
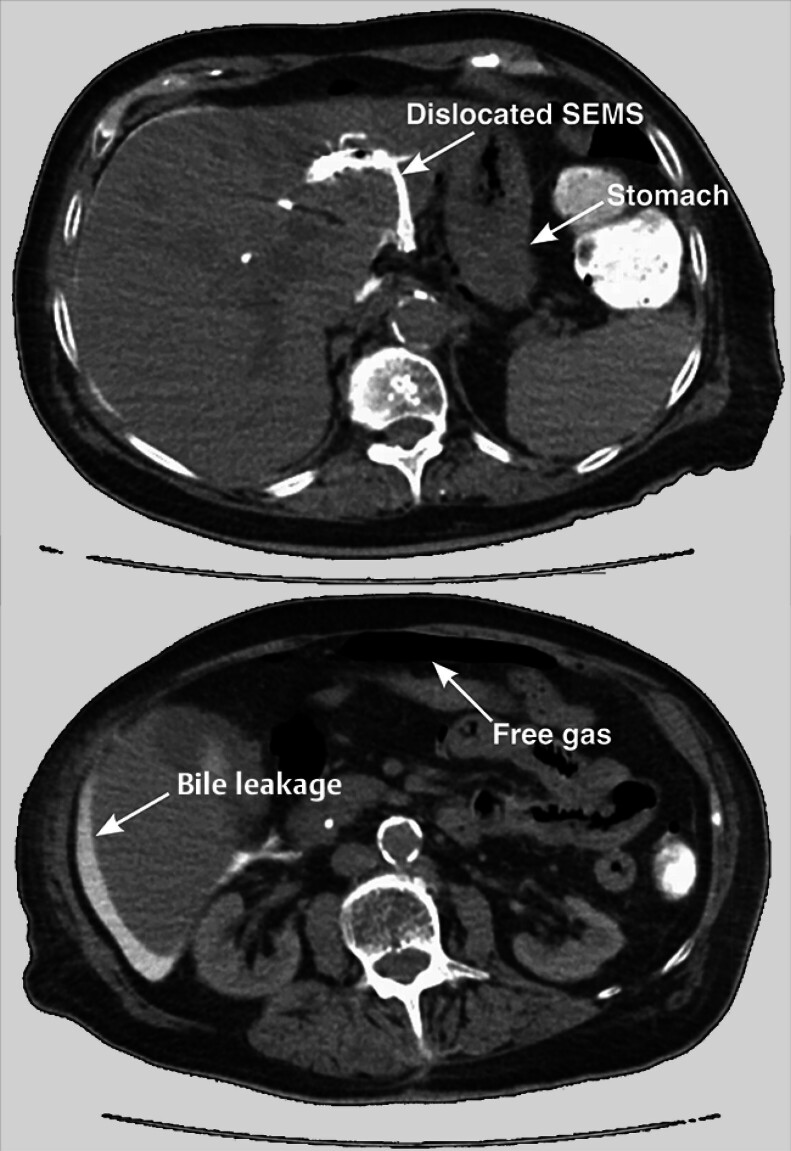
Computed tomography scan showed a dislocated self-expanding metal stent from the endoscopic ultrasound-guided hepaticogastrostomy (EUS-HGS), bile leakage, and pneumoperitoneum.

After careful interdisciplinary discussion, we performed endoscopic interventions under general anesthesia ( Video 1 ). The gastroscopy revealed a large perforation ( Fig. 3 ). The metal stent was endoscopically removed from the peritoneal cavity through the perforation using additional fluoroscopic guidance, a thin gastroscope, underwater endoscopy, and alligator grasping forceps to retrieve the metal stent ( Fig. 4 ). Subsequently, the perforation was successfully closed with through-the-scope-clips. A new EUS-HGS with a semi-covered metal stent was then performed to ensure adequate bile drainage ( Fig. 5 ). Gastroscopic follow-up confirmed proper closure of the esophageal perforation, correct placement of the metal stent, and sufficient bile drainage. The patient was extubated after the procedure and recovered well. Serum bilirubin levels decreased significantly and rapidly.

Endoscopic treatment of dislocated hepaticogastrostomy stent involving removal of a dislocated self-expanding metal stent via endoscopic laparoscopy, closure of the esophageal perforation with through-the-scope clips, and performance of a new endoscopic ultrasound-guided hepaticogastrostomy with a self-expanding metal stent.Video 1

**Fig. 3:**
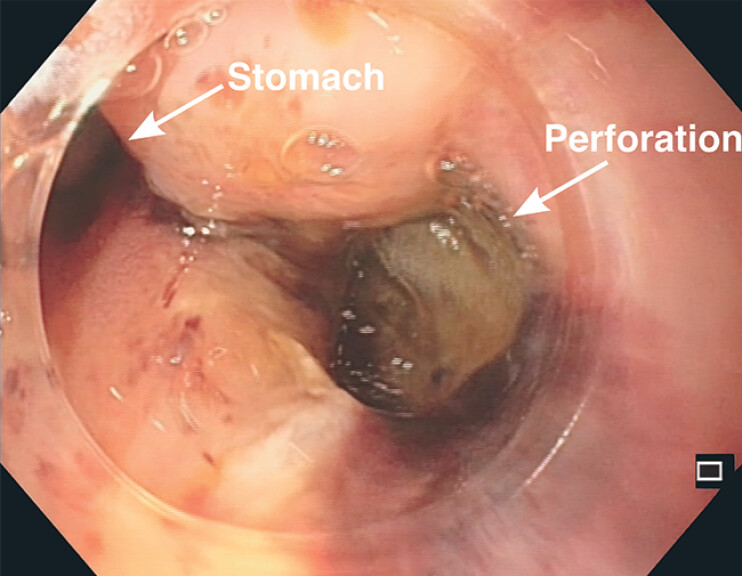
Gastroscopy confirmed a large perforation of the distal esophagus caused by the misplacement of the EUS-HGS stent.

**Fig. 4:**
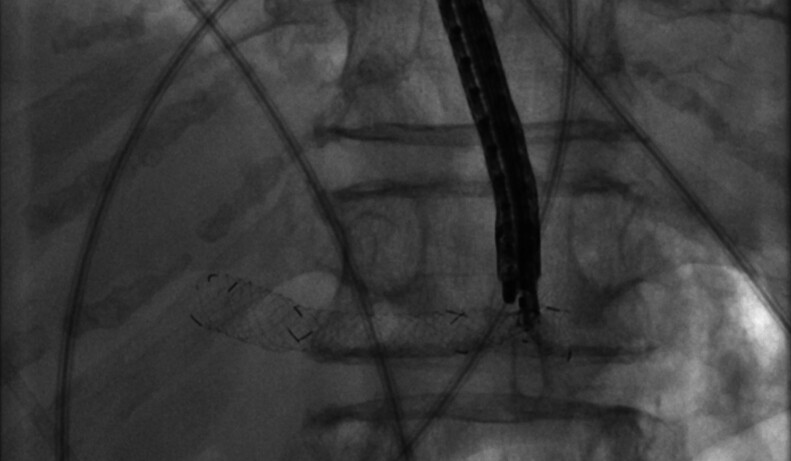
Dislocated metal stent was reached with a thin gastroscope using underwater endoscopy and grasped with alligator forceps to remove it through the perforation of the distal esophagus.

**Fig. 5:**
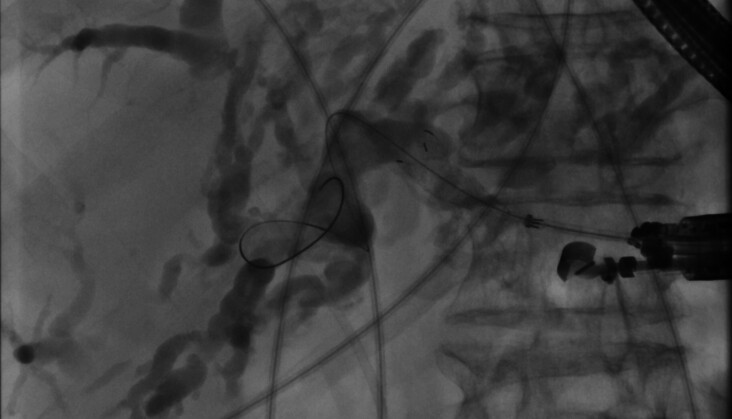
Successful placement of a new EUS-HGS stent, which led to adequate bile drainage.

This case demonstrates that dislocation of an EUS-guided drain into the peritoneal cavity with perforation of the gastrointestinal wall can be successfully treated endoscopically.

Endoscopy_UCTN_Code_CPL_1AL_2AD

## Notes

Endoscopy E-Videos https://eref.thieme.de/e-videos
*E-Videos* is an open access online section of the journal *Endoscopy* , reporting on interesting cases and new techniques in gastroenterological endoscopy. All papers include a high-quality video and are published with a Creative Commons CC-BY license. Endoscopy E-Videos qualify for HINARI discounts and waivers and eligibility is automatically checked during the submission process. We grant 100% waivers to articles whose corresponding authors are based in Group A countries and 50% waivers to those who are based in Group B countries as classified by Research4Life (see: https://www.research4life.org/access/eligibility/ ). This section has its own submission website at https://mc.manuscriptcentral.com/e-videos .
